# How personal and standardized coordination impact implementation of integrated care

**DOI:** 10.1186/s12913-015-1079-6

**Published:** 2015-10-02

**Authors:** Justin K. Benzer, Irene E. Cramer, James F. Burgess, David C. Mohr, Jennifer L. Sullivan, Martin P. Charns

**Affiliations:** 1 Center for Healthcare Organization and Implementation Research, VA Boston Healthcare System, 150 S. Huntington Ave (152-M), Boston, MA 02130 USA; 2 Department of Health Policy and Management, Boston University School of Public Health, 715 Albany Street Talbot Building, T2W, Boston, MA 02118 USA; 3 VISN 17 Center of Excellence for Research on Returning War Veterans, Waco, TX USA

**Keywords:** Primary care, Mental health, Integrated care, Integration, Coordination, Intervention implementation, Implementation science, Collaborative care

## Abstract

**Background:**

Integrating health care across specialized work units has the potential to lower costs and increase quality and access to mental health care. However, a key challenge for healthcare managers is how to develop policies, procedures, and practices that coordinate care across specialized units. The purpose of this study was to identify how organizational factors impacted coordination, and how to facilitate implementation of integrated care.

**Methods:**

Semi-structured interviews were conducted in August 2009 with 30 clinic leaders and 35 frontline staff who were recruited from a convenience sample of 16 primary care and mental health clinics across eight medical centers. Data were drawn from a management evaluation of primary care-mental health integration in the US Department of Veterans Affairs. To protect informant confidentiality, the institutional review board did not allow quotations.

**Results:**

Interviews identified antecedents of organizational coordination processes, and highlighted how these antecedents can impact the implementation of integrated care. Overall, implementing new workflow practices were reported to create conflicts with pre-existing standardized coordination processes. Personal coordination (i.e., interpersonal communication processes) between primary care leaders and staff was reported to be effective in overcoming these barriers both by working around standardized coordination barriers and modifying standardized procedures.

**Discussion:**

This study identifies challenges to integrated care that might be solved with attention to personal and standardized coordination. A key finding was that personal coordination both between primary care and mental health leaders and between frontline staff is important for resolving barriers related to integrated care implementation.

**Conclusion:**

Integrated care interventions can involve both new standardized procedures and adjustments to existing procedures. Aligning and integrating procedures between primary care and specialty care requires personal coordination amongst leaders. Interpersonal relationships should be strengthened between staff when personal connections are important for coordinating patient care across clinical settings.

## Background

Achieving integrated health care is an international priority [1–5]. However, achieving this promise requires healthcare managers to coordinate care effectively across an increasingly specialized healthcare workforce. The popularity of team-based health care is based in part on a widespread recognition of this fundamental problem, but does not resolve difficulties related to coordinating across specialized providers or units such as primary care and mental health [6–9]. A key challenge for healthcare managers is to develop policies, procedures, and practices that integrate care across these specialized units, and implement those changes into healthcare units’ existing social relations and processes [10].

Integration is commonly categorized as functional, physician-system, or clinical integration [11] (p.129). Integration of primary care and mental health is a type of clinical integration in that it includes structures and systems that coordinate medical and mental health services across people and over time [11–14]. Recent studies identified challenges in implementing integrated primary care and mental health. Difficulties include coordinating referrals [15], managing a wide range of appointment types [16], determining the timing and length of appointments [17], clarifying responsibilities between integrated versus specialty mental health clinics [18], and coordinating amongst providers [19]. This prior research demonstrates that organizational changes intended to integrate services between professions may not translate into either patient experiences of integrated care [20] or effective collaborative care among providers. The purpose of this study is to use the implementation of a policy to clinically integrate primary care and mental health to answer the following research questions:

1. How do organizational factors impact coordination between primary care and mental health?.
2. How can the implementation of integrated care be facilitated?.

### Conceptual framework

Drawing from the organization theory literature, coordination processes are key factors in managing interdependent tasks across professional and team/unit boundaries. Organizational coordination is defined as the integration of individuals or work units across an organization to facilitate the achievement of shared objectives [21]. This paper focuses on two modes of coordination, standardized and personal [22].

Standardized coordination consists of impersonal codified processes developed for interdependent tasks. A key characteristic of standardized coordination is that once developed, coordination is conducted with minimal interpersonal interactions. Standardized coordination can be implemented through organization processes, such as formal rules, designation of responsibilities, protocols and training. Standardized modes of coordination facilitate appropriate and efficient delivery of healthcare services by developing procedures to handle the most common coordination needs in an organization. For example, providers often have explicit criteria for when to conduct specific tests or when to refer to specialists. In addition to addressing other aspects of quality care, these standards focus on minimizing the work needed to coordinate between units. However, standardization processes are not effective for highly uncertain work or in situations where coordination needs are not well understood.

Personal coordination consists of interpersonal communication for interdependent tasks. Personal coordination can be formalized through scheduled meetings or requests for consultation. Personal coordination also can occur informally through communication among peers or communication with a person in a designated coordination role. Personal coordination can be used when coordination processes are not codified. In this way, personal coordination could be viewed as meeting patient needs and preferences that are not adequately addressed through existing standardized coordination processes. Personal coordination also can be used when there is uncertainty regarding which standardized coordination process is appropriate. For example, a primary care provider might call a psychiatrist on duty (i.e., a designated coordination role) to determine whether a patient’s depression symptoms warrant special attention. Personal coordination can be time consuming because it involves communication.

Some research indicates that both standardized and personal coordination are needed to optimize patient care [22]. However, there has been little research devoted towards understanding how standardized and personal coordination support integration across clinical units. The current study will examine standardized and personal coordination processes during a period when managers were implementing an intervention to integrate primary care and mental health services. This structural change necessitated revisions to standardized coordination processes.

## Methods

### Setting

The Department of Veterans Affairs (VA) is the largest healthcare system in the United States (U.S.). VA provides care to over 8 million Veterans. VA is organized into medical centers that employ salaried physicians. Medical centers typically provide primary care in both hospital-based and community-based outpatient clinics. VA medical centers have quasi-independent management structures and budgets that are distributed through a form of capitation. This system is different from other U.S. healthcare systems that reimburse physicians through fee-for-service. All sampled VA medical centers in this study have a service line management structure, where multi-disciplinary staff (physicians, nurses, therapists, social workers and clerical staff) report to service line managers in primary care, mental health, or other specialty areas.

### Intervention design

In 2008, VA mandated a transition from consultation to collaborative models of mental health care in the primary care setting. Communication and consultation is the traditional process whereby primary care providers consult with psychiatrists on an as needed basis [23]. Collaborative care is a process of shared responsibility and concurrent treatment between primary care and mental health providers [24]. Details of how to operationalize and implement collaboration were left to the discretion of local leaders.

The intervention dimension relevant to the current study is that all hospital-based and large outpatient clinics (more than 10,000 unique patients per year) were required by top VA managers to staff co-located mental health providers in primary care clinics in order to provide collaborative care for patients with short-term mental health care needs. Patients with long-term and complex needs would continue to be seen in the specialty mental health service. Medical centers were allowed to tailor this Primary Care/Mental Health Integration (PC/MHI) mandate to address local priorities. Many aspects of this national intervention have been described elsewhere [25, 26].

### Participants

Key informants included 30 clinic leaders and 35 frontline staff who were recruited from 16 primary care and mental health clinics across eight VA medical centers. The medical centers were a convenience sample. The eight medical centers were involved in a VA management evaluation of PC/MHI, and are reported here as a secondary analysis. The VA Boston IRB ruled that data from this evaluation study could be used without informed consent, but no quotations were allowed in order to protect informant confidentiality.

Interviews at each clinic included approximately one leader from each of primary care and mental health, one primary care physician, and one co-located psychologist, psychiatrist, social worker, or mental health nurse. All employees who were approached for interviews agreed to participate. Interviewees were drawn from a hospital-based clinic and up to two large freestanding outpatient clinics in each medical center. Large clinics were chosen because clinics with less than 10,000 unique patients were not required to have the same staffing in regard to mental health care as larger clinics.

### Data collection

Interviews were conducted during July and August 2009 as part of a managerial evaluation. Semi-structured 45-min interviews purposefully sampled primary care and mental health leaders who then identified staff who were knowledgeable regarding implementation. Telephone interviews were conducted with a note taker instructed to record responses as close to verbatim as possible. Voice recording was not conducted to protect informant confidentiality. Reporting was approved by the VA Boston Healthcare System institutional review board. Documentation of informed consent was not required. However, the institution review board did not allow the use of quotations in order to protect informant confidentiality.

Table 1 presents the seven questions and probes from the semi-structured interview guide. Interviewers first asked “grand tour” questions that allowed informants to describe the current and evolved processes of care without a researcher-imposed framework. Informants then described the coordination concept from their perspectives. Later questions targeted current coordination deficits, limits of coordination, psychological barriers between clinics, and interpersonal coordination.

**Table 1:** Interview questions and specific concepts

Interview Question	Specific Concepts
1. Imagine that a patient with depression symptoms comes to the clinic. Can you walk me through a typical process of care?	Referral process, differences between diagnoses
2. How has this process changed over the past 10 years? (or since you arrived in the clinic)?	Recent changes, leadership support, referrals, interpersonal interactions, physical structure
3. Tell me about your sense of the need for coordination between primary care and mental health.	Examples of good and poor coordination
4. How would you change your clinic to better coordinate care?	Communication, collaboration, resource barriers
5. Have you or anyone you know had to develop your own coordination procedures to ensure that patients receive the best care?	Work-arounds, ad-hoc coordination procedures
6. Can you tell me about the relationship between the people in the primary care and mental health clinics?	Face to face contact, trust
7. In what situations would you say that teamwork is most important?	Co-workers back each other up

Note. Each interview question was phrased broadly to allow for participants to respond without imposing a framework upon them. If the responses did not relate to the specific concepts, then the interviewer probed more deeply into those areas

### Data analysis

**Fig. 1:**
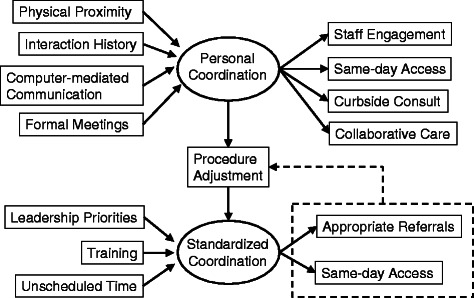
Organizational process antecedents and outcomes of personal and standardized coordination for integrated mental health care. Relationships were suggested by key informant interviews. Dashed arrow indicates that the discrepancies between the current and ideal state of standardized coordination may result in procedure adjustment if supportive personal coordination is present between leaders of different services and/or between frontline staff

The analysis focused on personal and standardized coordination as sensitizing concepts from the conceptual framework, but was open to related codes [27]. Our initial inductive coding closely represented the interview data. The purpose of these provisional codes was to reduce the complexity of the data while closely representing informants’ perspectives [27]. From these provisional codes, the authors developed a codebook of 18 emergent concepts using the constant comparative method (i.e., comparing statements for each code within the same interview as well as across interviews). Figure 1 includes seven codes about organizational factors impacted coordination (left side of figure), and six codes about how coordination impacted integrated care (right side of figure). Four codes reflected how implementing the new coordination procedures affected organizational factors and are discussed in more detail below (i.e., gatekeeping, optimizing processes, expanding services, and short-term work-arounds). One author then coded incident-by-incident to identify types of organizational factors that informants discussed in relation to coordination. A second author used this codebook to recode all of the interview data. The authors resolved all disagreements through discussion.

The entire group of authors met to evaluate whether each quotation represented good or poor instances of personal or standardized coordination. In these meetings, data were coded as personal or standardized coordination, but data also included emergent concepts (i.e., organizational factors) from the prior analyses. The first author thematically integrated the two sets of analyses by comparing between informants in a site, and also between sites to identify how informants reported that organizational factors impacted coordination and patient care. Because IRB restrictions preclude the use of quotations, illustrative examples are summarized rather than quoted.

## Results

Most clinics were transitioning to integrated care (15 of 16; 1 clinic planning) with no clinics reporting fully implemented integrated processes. The concepts of standardized and personal coordination were well represented across the clinics (149 quotations). Reports suggested that most clinics have good personal coordination processes (10 good, 2 poor, 4 neither/unclear), and clinics appeared to be split regarding standardized coordination processes (6 good, 7 poor, 3 neither/unclear).

Figure 1 summarizes the thematic relationships for each of personal and standardized coordination. The dotted line in Fig. 1 presents a feedback loop for standardized coordination procedures. As described in more detail below, there were ten clinics where personal coordination was reported to impact standardized coordination through a feedback loop, such that interpersonal interactions between services were used to adjust standardized coordination procedures to better serve patient and provider needs. Table 2 defines the organizational concepts and presents the hypothetical processes through which they were reported to impact integrated care.

**Table 2:** Organizational concepts related to coordination between primary care and mental health

Organizational Concept	Definition	Potential Impact on Integrated Care
Physical proximity	Distance between the offices of primary care and mental health providers	Promote staff engagement and curbside consults by increasing familiarity
Interaction history	The degree to which primary care and mental health providers have established cooperative relationships	Facilitate curbside consults and same-day access when physical proximity is not present.
Formal meetings	Inclusion of mental health providers in regularly scheduled primary care meetings	Promote staff engagement by increasing familiarity and communicate regarding patient treatment status
Computer-mediated Communication	Use of an “additional signer” process to communicate between providers.	Communicate regarding patient treatment status
Leadership priorities	Differences in mission and values between primary care and mental health	Limit integration of referral processes
Training	Training to standardize referral procedures Training to standardize skills that support interactions between primary care and mental health	Tailor referrals to needs of both primary care and mental health Increase flexibility in standardized procedures by increasing the number of staff who can complete tasks related to integrated care
Unscheduled time	Time slots left open for unanticipated patient needs	Flexibility in standardized procedures allows for curbside consults and same-day access

### How do organizational factors impact personal coordination?

#### Physical proximity

Informants at 11 clinics suggested that physical proximity between mental health staff and primary care staff impacted staff engagement and curbside consults. For example, a psychologist at one clinic indicated that physical proximity helped build familiarity and engagement in integrated care. According to this psychologist the number of consults seemed to increase after eating in the lunch room with the primary care providers and discussing the types of assistance that mental health could provide for their patients. Primary care providers in seven clinics with physical proximity reported being able to knock on mental health providers’ doors to request a quick curbside consult or to introduce a patient to the mental health provider.

#### Interaction history

Some informants noted that physical proximity was helpful but not necessary for promoting personal coordination. Instead, a history of positive interactions between professional colleagues may facilitate personalized, same-day treatment. In three clinics, a history of positive interactions between primary care and mental health was reported to promote telephone-based curbside consults. For example, one physician reported facilitating same-day access to substance abuse treatment by calling a colleague. The provider noted that because the patient was currently motivated for treatment, it was important to take advantage of this motivation.

#### Computer-mediated communication

Primary care providers in four clinics reported conducting collaborative care through the electronic medical record. Feedback was provided both passively by recording notes in the medical record and also actively by using an “additional signer” process in which primary care and mental health providers electronically certify that they reviewed patient notes. These reports suggest that personal coordination can facilitate collaborative care, whether the communication is formal or informal.

#### Formal Meetings

Personal coordination also occurred through formal structures. Managers and frontline staff in three different clinics reported how formal meetings were used to increase engagement of primary care staff in integrated care. One co-located psychologist reported how the meeting provided an opportunity to “sell” collaborative care. Informants at the two other clinics reported that formal meetings were used to communicate between primary care and mental health about patients’ treatment status.

### How do organizational factors impact standardized coordination?

#### Service priorities

Differences in priorities and goals between primary care and mental health created problems for integrating referral procedures between the two services. For example, at one clinic, both the primary care provider and integrated mental health provider reported conflicts between the integrated mental health staff and the psychiatrist on duty regarding their definition of an appropriate referral. These key informants reported that referrals to mental health for Axis II (i.e., personality disorder) patients were commonly rejected. This decision serves mental health goals of treating PTSD and other serious mental illnesses, but does not consider primary care goals of managing a very challenging patient population. In a related example, mental health procedures in one clinic required that substance abuse treatment precede mental health care. This reflects mental health goals of ensuring that patient referrals are easier to manage (because the mental health problems would be clearer without co-occurring substance abuse). A consequence was that primary care became the default mental health provider for patients who refused substance abuse treatment unless the disorder was intentionally omitted from the mental health consult. In third example, a primary care informant reported that the electronic consult system would reject depression consults to mental health if providers did not indicate that patients had received the maximum dose of antidepressants, regardless of patient preferences. This reflects a mental health goal of restricting referrals to the most severe cases of depression. However, this policy does not value primary care input into the decision-making. These types of differences between primary care and mental health in treatment priorities impacted the degree to which primary care and mental health could be integrated.

#### Training

Training in same day access procedures was reported as important by mental health providers in two clinics. In these clinics, same day referrals were not communicated from the primary care provider to the mental health provider. Training clerks to facilitate this process was reported to improve same-day access. Providers in three other clinics reported training providers about the new electronic consult system procedures in order to ensure that patients were referred to the appropriate providers.

#### Unscheduled time

Unscheduled time promoted same-day access. Primary care informants in two clinics reported that reserving time for unscheduled consults facilitated same-day access by allowing flexibility in standardized procedures. According to these informants, flexibility in standardized scheduling facilitated personal coordination for impromptu curbside consults and brief same-day encounters.

### How can the implementation of integrated care be facilitated?

Analyses highlight the importance of personal coordination in the implementation of integrated care. Notably, personal coordination is not the same as physical proximity. For example, informants in three sites reported how a lack of available space limited co-location. Two of those sites reported limited cross-service collaboration (i.e., personal coordination). One informant characterized the space negotiations as a “nasty” conflict. Both sites also reported conflicts in their standardized coordination referral practices. In contrast, the third site also experienced space barriers, but informants reported that barriers were resolved through negotiations between primary care and mental health leaders (i.e., personal coordination). Both primary care and mental health staff at this site reported positive examples of collaboration.

Personal coordination among primary care and mental health leaders was reported to impact the implementation of integrated care. For example, a co-located psychologist reported that personal coordination between primary care and mental health leaders decreased gatekeeping. That is, specialty mental health previously had canceled consults from primary care due to restrictive standardized inclusion criteria for specialty treatment. Personal coordination in the form of negotiation between primary care and mental health leaders resolved the conflict by determining that those consults were appropriate. In another example, primary care and mental health leaders both reported a conflict with the Emergency Department (ED) over which service should handle urgent mental health concerns. The primary care leader reported that prior to PC/MHI implementation the ED would handle urgent mental health issues. After PC/MHI implementation, mental health providers in the ED believed that the PC/MHI program should take on that responsibility. However, primary care and PC/MHI providers did not perceive that urgent mental health care was within the scope of integrated care. Personal coordination in the form of negotiation among primary care and mental health leaders was used to resolve this conflict and modify the standardized procedures.

Personal coordination among front line staff was reported to generate short-term solutions to standardized barriers. For example, a primary care provider reported how one patient with a severe psychiatric condition could not access long-term care. The physician was able to obtain placement for this patient by leveraging a personal relationship with a psychiatrist. Using a similar short-term orientation, one mental health provider reported managing all of the primary care consults for PTSD. The rationale was that the PTSD clinic is overworked and patients often have co-morbidities, such as substance abuse and social issues that can be resolved before involving the PTSD clinic. These reports indicate that personal coordination may be used for short-term fixes to standardized coordination barriers such as gatekeeping and misaligned procedures. However, as presented above, our data also indicates that it also is possible to use personal coordination for larger-scale process redesigns to address the root causes of coordination conflicts.

Personal coordination was also reported by front line staff to be useful for improving processes that impact the intervention. For example, a co-located mental health provider reported that one clinic used monthly joint meetings to voice problems regarding standardized electronic referral system. The clinic used meetings to standardize the intake process and advise providers regarding the appropriate types of referrals to mental health. Personal coordination was also used to identify how the integrated care program could be expanded in order to address priorities for both primary care and mental health. Informants in three clinics reported that communication between primary care and mental health leaders resulted in developing additional services that primary care providers perceived were important such as focusing on patient adherence to chronic care guidelines and pain management. According to one co-located psychologist, an increased focus on services relevant to primary care increased primary care provider receptivity to conducting screening and management of mental health patients that had been perceived as being the responsibility of mental health.

The analyses summarized above suggest a feedback loop (see Fig. 1) where implementation barriers could be resolved over time by personal coordination among both leaders and frontline staff. Feedback loops are hypothetical paths whereby a discrepancy between a current state and a perceived ideal state is modified by a specific process or mechanism [28]. Informants reported discrepancies in how standardized processes were not aligned with the needs of primary care and mental health staff, as well as patients. Promoting personal coordination can facilitate process re-alignment.

## Discussion

This study identifies challenges to integrated care that might be solved with attention to personal and standardized coordination. A key finding was that personal coordination both between primary care and mental health leaders and between frontline staff is important for resolving barriers related to integrated care implementation. We conducted an iterative process of comparisons across sites, and have highlighted the factors that were repeated in different clinics. Thus, we have reasonable confidence that findings may be transferable to other settings.

This study advances the literature on clinical integration and coordination in primary care [6, 11, 29–33]. Coordination is an important dimension of primary care practice [34]. The current study reveals the challenges in implementing new procedures that conflict with or redefine existing standardized coordination practices. Healthcare managers seeking to implement integrated care interventions should be aware that existing standardized coordination procedures may create barriers to the new practices that are being implemented. Personal coordination among both leaders and frontline staff may be necessary to overcome implementation barriers.

### Limitations

This study was designed to understand how coordination was being implemented in integrated care. As such, we did not seek out to collect data about what leaders did to overcome coordination barriers, or how practices may impact the effectiveness of integrated care. This study should be viewed as hypothesis generative, rather than providing definitive answers as to what leaders should do to implement integrated care.

Conducting the study during the initial phases of the implementation was important, as negotiations between services (i.e., personal coordination) over time may decrease pre-existing standardized barriers to care by modifying tasks and processes. Indeed, earlier quantitative research that focused on surgical services [22] did not target a specific intervention and found no evidence for negative effects of standardized coordination on patient care, but instead found that clinics with both personal and standardized coordination modes had higher levels of quality. Results of the current study elaborate this previous empirical work by showing that interventions can cause changes that may limit the utility of standardized procedures or cause a disconnect between standardized procedures and patient needs that can be addressed by personal coordination.

The setting for this is the VA, a somewhat unique healthcare system in the U.S. healthcare sector. However, we assert that standardized procedures may create implementation barriers during organizational coordination interventions in all types of healthcare facilities. Some of the specific barriers observed in this study may not be present in other U.S. healthcare systems or community-based practices. For example, we observed gatekeeping behaviors where referrals that did not conform to strict inclusion/exclusion criteria were refused. This would likely not be an issue in a system with a fee-for-service payment model. However, many of the findings in the current study may be transferable to fee-for-service healthcare organizations.

### Future research directions

Patients, particularly those with multiple chronic conditions, require care across multiple healthcare professionals and settings. The PC/MHI intervention promoted co-located collaborative mental health care, but it is important to consider how coordination might be enhanced in situations where healthcare workers are geographically distributed. That is, how can interdisciplinary collaborative teams be designed if the team members are not co-located?.

Leadership is an organizational process that can bridge these geographical gulfs [35]. The current study demonstrated how leaders can facilitate intervention implementation by resolving conflicts among standardized procedures, and generates some hypotheses regarding best practices for implementation. Identifying management factors that can improve collaborations (e.g., personality and individual differences [36]) across distributed contexts is a cutting-edge topic in the organization sciences. Thus the current paper cannot provide clear guidance but identifies the coordination of geographically separated healthcare services, and the role of individual differences in personal coordination as important areas of future research.

## Conclusion

Achieving the promise of integrated care requires coordination across specialized work units. As healthcare managers face these coordination challenges, we recommend that personal coordination be promoted between leaders of these specialized units. Specifically, following Table 2, we recommend (1) promoting interactions among staff from specialized units to increase familiarity and develop a shared history of positive interactions, and (2) promoting interactions among leaders of specialized units to develop a shared mission and values regarding integrated care. Personal coordination can facilitate tailoring of standardized coordination procedures to be better aligned with patient mental health and medical care needs. However, because personal coordination requires more time and effort than standardized processes, coordination should be standardized whenever possible. Thus, following Table 2, we also recommend that leaders involve all relevant stakeholders in identifying potential problems with existing standardized processes, revise the processes, and look for opportunities to standardize effective processes through training and other improvement activities to engage with front line providers.

## Abbreviations

VA: Department of veterans affairs
PC/MHI: Primary care/mental health integration
